# Is ovarian reserve reduction following endometriotic cystectomy predicted? The implication for fertility preservation counseling

**DOI:** 10.3389/fendo.2022.996531

**Published:** 2022-09-21

**Authors:** Johnny S. Younis, Nora Shapso, Ido Izhaki

**Affiliations:** ^1^ Reproductive Medicine, Department of Obstetrics and Gynecology, Baruch-Padeh Medical Center, Poriya, Israel; ^2^ Azrieili Faculty of Medicine, Bar-Ilan University, Safed, Israel; ^3^ Department of Evolutionary and Environmental Biology, University of Haifa, Haifa, Israel

**Keywords:** endometriosis, endometrioma, fertility preservation, anti-Müllerian hormone, endometriotic cystectomy

## Introduction

Ovarian reserve depletion secondary to an intact endometrioma continues to be actively debated (1, 2) and has been recently challenged (3). Conversely, endometriotic cyst surgery, specifically endometriotic ovarian cystectomy, seems to have an irreversible damaging impact and is of concern for women and practitioners alike. Past histological studies have demonstrated inadvertent primordial follicle removal adjacent to the endometrioma, which seems inevitable even in experienced hands (4, 5). Furthermore, well-performed systematic reviews and meta-analyses have resulted in a significant irreversible reduction in serum AMH levels (3, 6), suggesting potentially lasting damage to the reproductive life span.

Recently, our group has shown a significant drop in serum AMH levels by 1.65 ng/ml (95% CI: 1.15 to 2.15) and by 2.03 ng/mL (95% CI: 1.47 to 2.58) at 9-12 months postoperatively as compared to baseline in the unilateral and bilateral ovarian endometriotic cystectomy groups, respectively, corresponding to 39% and 57% decline of the functional ovarian reserve following surgery (3). Furthermore, we have shown that AMH is a much more sensitive biomarker than AFC in this setting (7). Since our publication considered studies that employed the stripping technique (3), more conservative and less invasive modalities of endometrioma treatment impact on ovarian reserves, such as ultrasound-guided sclerotherapy or laser vaporization, ought to be further explored (8, 9).

The impact of endometrioma cystectomy on ovarian reserve argues that these women need fertility preservation counseling. However, the main question remains whether fertility preservation should be recommended to every woman with ovarian endometriosis considering surgery and planning for a future pregnancy and whether preoperative serum AMH levels are essential for adopting this strategy.

## Fertility preservation in ovarian endometriosis

At present, many papers suggest fertility preservation, particularly oocyte vitrification, in women with intact endometrioma considering surgery and postponement of pregnancy. Interestingly, most of these publications are opinion papers and experts’ viewpoints. At the same time, only a few cohort studies of actual oocyte vitrification in women with endometriosis have been published (10–16). Although ovarian tissue cryopreservation is suggested in this setting, only a few case reports have been published so far (17). The utility of such an approach in this setting needs further assessment.

Overall, although the true benefit of fertility preservation in women with endometriosis remains unknown, the recent guidelines of the ESHRE endometriosis core group recommend discussing the pros and cons of such practice, especially in women with extensive ovarian endometriosis (18).

Among published papers on oocyte vitrification in women with endometriosis, all retrospectively conducted (**Table 1**), the most comprehensive so far is from Spain (12). It contains 1044 women with intact endometrioma that underwent oocyte preservation, of which 43% came back to thaw their gametes to attain pregnancy. The main findings of this study suggest oocyte preservation before 35 years of age and before endometriotic surgery to obtain significantly better oocyte yield and cumulative live birth rate (CLBR) (12). A recent report by a different group from France (n = 146) corroborates these findings and advocates integrating fertility preservation into endometriosis management (16).

**Table 1:** Fertility preservation (Oocyte Vitrification) in Cases with Endometriosis: Summary of the Literature.

Study	Publication site	Country	Design	Number of women	Women returned
Garcia-Velasco et al., 2013 (10)	Fertil Steril	Spain	Retrospective multicenter observational	38	5
Raad et al., 2018 (11)	J Obstet Gynecol Reprod Biol	France	Retrospective observational cohort	70	ND
Cobo et al., 2020^#^ (12)	Fertil Steril	Spain	Retrospective observational cohort	1044	485
Kim et al., 2020 (13)	RBMOnline	S. Korea	Retrospective cohort	35	ND
Cobo et al., 2021^#^ (14)	RBMOnline	Spain	Retrospective observational cohort	1044	485
Hong et al., 2021* (15)	Fron Endocrinol	S. Korea	Retrospective cohort	62	ND
Santulli et al., 2021* (16)	RBMOnline	France	Retrospective observational cohort	146	ND

*Including embryo freezing.

^#^Same cohort.

ND, not disclosed.

## The magnitude of surgery’s impact on ovarian reserve is unpredictable

The relevance of preoperative serum AMH levels in predicting the likelihood of pregnancy attainment or the need for fertility preservation is unclear since studies reporting on this topic have made inconsistent conclusions (18).

Counseling for oocyte preservation in women with ovarian endometriosis may depend on several composite and interrelated factors: age, preoperative serum AMH level, endometrioma diameter and laterality, uni-or multicystic display, pelvic anatomy and adhesions, surgical skills, techniques, and complexity, hemostasis methods, and previous surgery. While some of these factors may be readily assessed before surgery, others would not.

While the decrease in functional ovarian reserve following endometriotic cystectomy is exemplified by statistically significant serum AMH levels reduction, the range in AMH reduction among individual-operated women seems vast. Some women may exhibit a substantial serum AMH reduction, while others may show a minor decrease in serum AMH levels. In many cases, the magnitude of serum AMH decrease is unpredictable. Nowadays, there is no means to predict individual AMH reduction since many interrelated measurable and non-measurable confounders are involved.

Extreme damage to ovarian reserve, causing premature ovarian insufficiency, has been shown at an advanced reproductive age following bilateral endometriotic cystectomy and repeat surgery (19–21). However, it occurs only in 2.4% of cases (19), while the impact on the ovarian reserve is unanticipated in other women.

To quantify the wide variation of AMH reduction following endometriotic cystectomy, we have looked again into eligible studies in our recent systematic review examining the impact of endometriotic cystectomy on serum AMH levels (3). Twelve studies were eligible for meta-analysis, including 783 women: 489 and 294 in the unilateral and bilateral groups, respectively. This time, we calculated the coefficient of variation (CV) in serum AMH reduction following endometriotic cystectomy at the early (1 – 4 weeks), intermediate (6 weeks – 6 months), and late (9 – 12 months) postoperative periods. The CV is the ratio of the standard deviation to the mean difference of serum AMH values. The higher the coefficient of variation, the greater the level of dispersion around the mean, suggesting a wide variation of postoperative serum AMH reduction. The CV among unilateral and bilateral groups in all three time periods, except in the late bilateral group, was substantial, ranging from 35% to 73% (**Table 2**), implying a vast and unpredictable span of serum AMH reduction following surgery. The modest CV in the bilateral cases during the late postoperative period is not surprising. There is an agreement that bilateral endometriotic cystectomy is constantly associated with significant harm to the ovarian reserve (22–24). Collectively, these results imply that whatever the pre-surgical serum AMH levels, the variation in post-surgical AMH levels reduction is vast, rendering them from being an obligatory requisite for fertility preservation counseling.

**Table 2:** Weighted mean difference, standard deviation (SD), and coefficient of variation (CV)* between preoperative and postoperative serum AMH levels in the unilateral and bilateral endometriotic cystectomy groups at the early, intermediate, and late postoperative periods.

	Weighted Mean DifferencePre- minus Postoperative ng/mL	SD	CV
Unilateral cystectomy			
1 – 4 weeks	1.38	0.88	64%
6 weeks – 6 months	0.92	0.67	73%
9 - 12 months	1.52	0.53	35%
Bilateral cystectomy			
1 – 4 weeks	2.11	1.34	64%
6 weeks – 6 months	1.50	1.07	71%
9 - 12 months	2.73	0.29	11%

*The coefficient of variation (CV) is the ratio of the standard deviation to the mean. The higher the CV, the greater the level of dispersion around the mean. It is expressed as a percentage.

## Serum AMH levels and live-birth prediction

Although serum AMH level is a robust biomarker of ovarian reserve, its reliability in live-birth prediction in the ART setting is poor (25). Furthermore, age was considered a better predictor than AMH for live-birth achievement (26). Recently, in a nationwide high-order study with a validated database, serum AMH levels were highly correlated with the CLBR in women with low ovarian reserve (AMH < 1 ng/mL) (27). This was mainly related to its association with the quantitative ART cycle outcomes, primarily the number of oocytes retrieved. Furthermore, AMH and female age each independently provided prognostic information regarding the probability of CLBR. Higher AMH levels were associated with greater CLBR for each age category. In women < 35 years of age, the CLBR ranged between 22.1% and 41.2%, stratified with AMH levels in-between < 0.1 and 0.91-1.0 ng/mL, respectively. In contrast, in women 41-42 years of age, the CLBR ranged between 6.1% and 11.1%, stratified with the same AMH levels (27). Accordingly, for women with intact endometrioma with serum AMH levels < 1.0 ng/mL, considering ovarian surgery and planning for future pregnancy, fertility preservation counseling should not be precluded, especially if they are < 35 years of age.

## Conclusion

There are no guidelines for fertility preservation in women with endometriosis, especially in women with intact endometrioma, planning for a future pregnancy. Furthermore, the true benefit of fertility preservation in young women with endometriosis remains unknown. Nonetheless, the recent guidelines of the ESHRE endometriosis group recommend discussing the pros and cons of such practice. The topic of fertility preservation in women with endometriosis is evolving, and only a few cohort studies have been published. Although there is a consensus on the harmful impact of endometriotic cystectomy on ovarian reserve, the effect of endometrioma per se on the quantitative and qualitative aspects of the ovarian reserve is still debated (1, 2, 28) and has been recently challenged (3, 29). The present opinion focuses on preoperative AMH levels and whether to be included in counseling women undergoing endometriotic cystectomy, specifically in those with low serum AMH levels.

There is a general agreement that age, endometrioma bilaterality, and previous ovarian surgery are key risk factors for significant serum AMH reduction and the threat of premature ovarian insufficiency in women undergoing endometriotic cystectomy. In most other cases, the degree of AMH reduction following surgery is unpredictable.

Serum AMH levels are reliable measures of functional ovarian reserve, essential for the patient’s counseling, and a good reference for future management, particularly in women postponing pregnancy. However, for women undergoing endometriotic cystectomy, preoperative AMH values do not predict surgical damage to the ovarian reserve. They should not be considered a critical measure to counsel fertility preservation before surgery. After surgery, serum AMH drop has a wide variation through a composite occurrence with numerous case-by-case interrelated factors that are largely unmeasurable and unpredictable. Until developing a reliable measure or score capable of predicting individual postoperative serum AMH reduction, discussing the pros and cons of fertility preservation with every woman planning a pregnancy regardless of her preoperative AMH levels seems plausible. Even in cases with low preoperative serum AMH levels (< 1 ng/mL), practitioners should not avert fertility preservation counseling, since reasonable CLBR may be achieved, notably in women below 35. Such a policy’s economic and psychological implications need further evaluation.

## Author contributions

JSY contributed to the conception and design of the manuscript and drafted the article. All authors contributed to data acquisition, analyses, and interpretation and approved the paper.

## Conflict of interest

The authors declare that the research was conducted in the absence of any commercial or financial relationships that could be construed as a potential conflict of interest.

## Publisher’s note

All claims expressed in this article are solely those of the authors and do not necessarily represent those of their affiliated organizations, or those of the publisher, the editors and the reviewers. Any product that may be evaluated in this article, or claim that may be made by its manufacturer, is not guaranteed or endorsed by the publisher.

## References

[1] Benaglia L Castiglioni M Paffoni A Sarais V Vercellini P Somigliana E . Eur is endometrioma-associated damage to ovarian reserve progressive? Insights from IVF cycles. J Obstet Gynecol Reprod Biol (2017) 217:101–5. doi: 10.1016/j.ejogrb.2017.08.034 28881264

[2] Kasapoglu I Ata B Uyaniklar O Seyhan A Orhan A Yildiz Oguz S . Endometrioma-related reduction in ovarian reserve (ERROR): A prospective longitudinal study. Fertil Steril (2018) 110:122–7. doi: 10.1016/j.fertnstert.2018.03.015 29935810

[3] Younis JS Shapso N Fleming R Ben-Shlomo I Izhaki I . Impact of unilateral versus bilateral ovarian endometriotic cystectomy on ovarian reserve: A systematic review and meta-analysis. Hum Reprod Update (2019) 25:375–91. doi: 10.1093/humupd/dmy049 30715359

[4] Roman H Tarta O Pura I Opris I Bourdel N Marpeau L . Direct proportional relationship between endometrioma size and ovarian parenchyma inadvertently removed during cystectomy, and its implication on the management of enlarged endometriomas. Hum Reprod (2010) 25:1428–32. doi: 10.1093/humrep/deq069 20378613

[5] Muzii L Marana R Angioli R Bianchi A Cucinella G Vignali M . Histologic analysis of specimens from laparascopic endometrioma excision performed by different surgeons: Does the surgeon matter? Fertil Steril (2011) 95:2116–9. doi: 10.1016/j.fertnstert.2011.02.034 21411079

[6] Raffi F Metwally M Amer S . The impact of excision of ovarian endometrioma on ovarian reserve: A systematic review and meta-analysis. J Clin Endocrinol Metab (2012) 97:3146–54. doi: 10.1210/jc.2012-1558 22723324

[7] Younis JS Shapso N Ben-Sira Y Nelson SM Izhaki I . Endometrioma surgery-a systematic review and meta-analysis of the effect on antral follicle count and anti-müllerian hormone. Am J Obstet Gynecol (2022) 226:33–51.e7. doi: 10.1016/j.ajog.2021.06.102 34265271

[8] Candiani M Ottolina J Posadzka E Ferrari S Castellano LM Tandoi I . Assessment of ovarian reserve after cystectomy versus ‘one-step’ laser vaporization in the treatment of ovarian endometrioma: a small randomized clinical trial. Hum Reprod (2018) 33:2205–11. doi: 10.1093/humrep/dey305 PMC6238368 30299482

[9] Kim GH Kim PH Shin JH Nam IC Chu HH Ko HK . Ultrasound-guided sclerotherapy for the treatment of ovarian endometrioma: An updated systematic review and meta-analysis. Eur Radiol (2022) 32:1726–37. doi: 10.1007/s00330-021-08270-5 34580747

[10] Garcia-Velasco JA Domingo J Cobo A Martínez M Carmona L Pellicer A . Five years’ experience using oocyte vitrification to preserve fertility for medical and nonmedical indications. Fertil Steril (2013) 99:1994–9. doi: 10.1016/j.fertnstert.2013.02.004 23465707

[11] Raad J Sonigo C Tran C Sifer C Durnerin IC Grynberg M . Oocyte vitrification for preserving fertility in patients with endometriosis: first observational cohort study and many unresolved questions. Letter to Editor Eur J Obstet Gynecol Reprod Biol (2018) 220:140–1. doi: 10.1016/j.ejogrb.2017.12.001 29221758

[12] Cobo A Giles J Paolelli S Pellicer A Remohí J García-Velasco JA . Oocyte vitrification for fertility preservation in women with endometriosis: an observational study. Fertil Steril (2020) 113:836–44. doi: 10.1016/j.fertnstert.2019.11.017 32145929

[13] Kim SJ Kim SK Lee JR Suh CS Kim SH . Oocyte cryopreservation for fertility preservation in women with ovarian endometriosis. Reprod BioMed Online (2020) 40:827–34. doi: 10.1016/j.rbmo.2020.01.028 32295746

[14] Cobo A Coello A de Los Santos MJ Giles J Pellicer A Remohí J . Number needed to freeze: Cumulative live birth rate after fertility preservation in women with endometriosis. Reprod BioMed Online (2021) 42:725–32. doi: 10.1016/j.rbmo.2020.12.013 33573907

[15] Hong YH Lee HK Kim SK Lee JR Suh CS . The significance of planned fertility preservation for women with endometrioma before an expected ovarian cystectomy. Front Endocrinol (Lausanne) (2021) 12:794117. doi: 10.3389/fendo.2021.794117 34975763 PMC8715896

[16] Santulli P Bourdon M Koutchinsky S Maignien C Marcellin L Maitrot-Mantelet L . Fertility preservation for patients affected by endometriosis should ideally be carried out before surgery. Reprod BioMed Online (2021) 43:853–63. doi: 10.1016/j.rbmo.2021.08.023 34649771

[17] Calagna G Della Corte L Giampaolino P Maranto M Perino A . Endometriosis and strategies of fertility preservation: A systematic review of the literature. Eur J Obstet Gynecol Reprod Biol (2020) 254:218–25. doi: 10.1016/j.ejogrb.2020.09.045 33011504

[18] Becker CM Bokor A Heikinheimo O Horne A Jansen F Kiesel L King K Kvaskoff M Nap A Petersen K Saridogan E Tomassetti C van Hanegem N Vulliemoz N Vermeulen N . ESHRE Endometriosis Guideline Group. Hum Reprod Open. (2022), (2):hoac009. doi: 10.1093/hropen/hoac009 35350465 PMC8951218

[19] Busacca M Riparini J Somigliana E Oggioni G Izzo S Vignali M . Postsurgical ovarian failure after laparoscopic excision of bilateral endometriomas. Am J Obstet Gynecol (2006) 195:421–5. doi: 10.1016/j.ajog.2006.03.064 16681984

[20] Coccia ME Rizzello F Mariani G Bulletti C Palagiano A Scarselli G . Ovarian surgery for bilateral endometriomas influences age at menopause. Hum Reprod (2011) 26:3000–7. doi: 10.1093/humrep/der286 21868401

[21] Takae S Kawamura K Sato Y Nishijima C Yoshioka N Sugishita Y . Analysis of late-onset ovarian insufficiency after ovarian surgery: Retrospective study with 75 patients of post-surgical ovarian insufficiency. PloS One (2014) 9:e98174. doi: 10.1371/journal.pone.0098174 24858999 PMC4032277

[22] Alborzi S Keramati P Younesi M Samsami A Dadras N . The impact of laparoscopic cystectomy on ovarian reserve in patients with unilateral and bilateral endometriomas. Fertil Steril (2014) 101:427–34. doi: 10.1016/j.fertnstert.2013.10.019 24269044

[23] Shao MJ Hu M He YQ Xu XJ . AMH trend after laparoscopic cystectomy and ovarian suturing in patients with endometriomas. Arch Gynecol Obstet (2016) 293:1049–52. doi: 10.1007/s00404-015-3926-4 26525696

[24] Kovačević VM Anđelić LM Mitrović Jovanović A . Changes in serum antimüllerian hormone levels in patients 6 and 12 months after endometrioma stripping surgery. Fertil Steril (2018) 110:1173–80. doi: 10.1016/j.fertnstert.2018.07.019 30396562

[25] Iliodromiti S Kelsey TW Wu O Anderson RA Nelson SM . The predictive accuracy of anti-müllerian hormone for live birth after assisted conception: a systematic review and meta-analysis of the literature. Hum Reprod Update (2014) 20:560–70. doi: 10.1093/humupd/dmu003 24532220

[26] Broer SL Broekmans FJ Laven JS Fauser BC . Anti-müllerian hormone: Ovarian reserve testing and its potential clinical implications. Hum Reprod Update (2014) 20:688–701. doi: 10.1093/humupd/dmu020 24821925

[27] Tal R Seifer DB Tal R Granger E Wantman E Tal O . AMH highly correlates with cumulative live birth rate in women with diminished ovarian reserve independent of age. J Clin Endocrinol Metab (2021) 106:2754–66. doi: 10.1210/clinem/dgab168 33729496

[28] Sanchez AM Viganò P Somigliana E Panina-Bordignon P Vercellini P Candiani M . The distinguishing cellular and molecular features of the endometriotic ovarian cyst: From pathophysiology to the potential endometrioma-mediated damage to the ovary. Hum Reprod Update (2014) 20:217–30. doi: 10.1093/humupd/dmt053 24129684

[29] Younis JS . Is oocyte quality impaired in cases with ovarian endometriosis? a second look into the clinical setting. Front Endocrinol (Lausanne) (2022) 13:921032. doi: 10.3389/fendo.2022.921032 35846314 PMC9279608

